# Optic Neuritis Presentation and Outcomes: A Single-Center Experience From Northern Saudi Arabia

**DOI:** 10.7759/cureus.81140

**Published:** 2025-03-25

**Authors:** Jluwi Almasaud, Madiha Mekni, Shog K Alahmed, Reema S Alanazi, Turki Alharbi

**Affiliations:** 1 Ophthalmology, King Khalid Hospital, Hail, SAU

**Keywords:** epidemiology, multiple sclerosis, neuromyelitis optica spectrum disorder, optic neuritis, saudi arabia

## Abstract

Objectives

This study aimed to analyze the demographic characteristics, clinical presentation, and treatment outcomes of patients with optic neuritis (ON) at King Khalid Hospital, Hail, Saudi Arabia.

Methods

A retrospective cohort study was conducted reviewing medical records of 40 patients diagnosed with ON between January 2021 and December 2024. Data collected included demographics, clinical presentations, neurological findings, imaging results, and treatment outcomes.

Results

The study population was predominantly female (n = 32, 80%), Saudi nationals (n = 33, 82.5%), and young adults aged 16-26 years (n = 24, 60%). Most cases were either idiopathic (n = 21, 52.5%) or associated with multiple sclerosis (MS) (n = 18, 45%), with a predominantly unilateral presentation (n = 37, 92.5%). Common symptoms included blurred vision (n = 38, 95.0%) and color vision changes (n = 31, 77.5%). Initial visual acuity was less than 20/200 in (n = 20, 50%) of cases. IV steroids were the primary treatment (n = 31, 77.5%), and (n = 32, 80%) of patients achieved vision better than 20/200 post-treatment. Significant associations were found between vision outcomes and both initial visual acuity (p = 0.011) and symptom duration (p = 0.041).

Conclusions

This study demonstrates generally favorable outcomes for patients with ON in the Hail region, with early presentation and treatment associated with better visual outcomes. The demographic and clinical patterns observed align with international literature while providing specific insights into the regional manifestation of the condition.

## Introduction

Optic neuritis (ON) is an acute inflammation of the optic nerve, presenting with a wide range of clinical features and underlying causes. It affects one to four people per 100,000 globally each year, with a higher prevalence among young, female, Caucasian individuals [1,2]. Although it can be idiopathic, ON is also linked to various demyelinating, inflammatory, infectious, or non-infectious etiologies, with multiple sclerosis (MS) being the most common, as reported in the literature [1].

The condition is commonly categorized into two major subtypes: typical and atypical ON. Typical ON is characterized by unilateral eye involvement, moderate visual loss, dyschromatopsia, and a favorable response to steroid therapy [3]. In contrast, atypical ON often involves bilateral eye involvement, more profound visual loss, and a poor response to treatment, making it more challenging to manage [4].

Clinically, ON presents with the classic triad of variable visual loss, periocular pain, and dyschromatopsia, and it may have typical or atypical presentations. Visual prognosis is usually good, depending mainly on the underlying cause [5].

To the best of our knowledge, few studies have focused on the specific clinical features and visual outcomes of this common condition in Middle Eastern populations, particularly within smaller tertiary centers in certain regions of Saudi Arabia, such as Hail [5,6].

Through this retrospective analysis of patients treated for ON at King Khalid General Hospital in Hail, we aim to uncover demographic and other specific factors that could shape the clinical presentation of ON and its visual outcomes in our community. This has the potential to significantly enhance patient care in the region, improving both short-term outcomes and long-term visual prognosis for those affected by this debilitating condition [7].

## Materials and methods

Study design

This retrospective cohort study was conducted at King Khalid Hospital, a leading tertiary care facility in Hail, Saudi Arabia, after approval by the Institutional Review Board at Hail Health Cluster (IRB Log Number: 2024-110). Patient records from January 2021 to December 2024 were reviewed to identify cases of new-onset ON. Cases were identified using the hospital's Oasis software by searching for diagnostic codes (International Classification of Diseases, Tenth Revision (ICD-10) coding) related to "optic neuritis," "multiple sclerosis," "retrobulbar neuritis," and "neuromyelitis optica." A manual review of medical records was also conducted to ensure comprehensive data collection. The final sample comprised 40 patients who met the inclusion criteria of a confirmed diagnosis of one of the specified optic nerve demyelinating or idiopathic disorders. 

Data collection

Data collected for each patient included demographic details, clinical presentation, results from neurological examinations, neuroimaging findings, visual acuity assessments, pupillary light reflex assessment to evaluate afferent pupillary defect, color vision testing using Ishihara color plates, slit-lamp biomicroscopy, dilated fundus examination, optical coherence tomography (OCT), and visual field assessment.

Inclusion criteria were (1) confirmed diagnosis of ON based on clinical presentation and ophthalmological examination; (2) age ≥16 years; (3) documented visual acuity data; and (4) brain MRI performed within two weeks of symptom onset.

Exclusion criteria were (1) alternative diagnoses explaining visual symptoms (ischemic, compressive, or infiltrative optic neuropathies); (2) incomplete follow-up data (<1 month); (3) pre-existing ocular conditions affecting visual assessment; and (4) traumatic optic neuropathy.

Case selection criteria

All diagnoses of ON were made by a neuro-ophthalmologist based on accepted diagnostic criteria for ON, which include clinical evaluation and neuroimaging findings. Patients with myelin oligodendrocyte glycoprotein antibody-associated disease (MOGAD) were not specifically excluded; however, none of the patients in our cohort tested positive for myelin oligodendrocyte glycoprotein (MOG)-IgG antibodies.

For neuromyelitis optica spectrum disorder (NMOSD), antibodies to aquaporin-4 (AQP4)-IgG serology were performed for suspected cases (bilateral involvement, severe visual loss, or poor recovery). Only one patient tested positive and was classified under NMOSD. The study included both first-time and recurrent ON presentations. Recurrent cases not related to MS were classified as idiopathic ON if no other etiology was identified. A minimum follow-up period of one month was required for inclusion in the study to ensure adequate assessment of treatment outcomes. Patients with incomplete follow-ups of less than one month were excluded. Cases were diagnosed by trained ophthalmologists, and we included a multidisciplinary review to validate the diagnoses.

Sample size justification

A formal power analysis was not conducted; however, the sample size of 40 patients was determined based on feasibility, the rarity of ON cases in our region, the limitations of a single-center study, and alignment with similar studies in the literature [5,6].

While the generalizability is limited, the results offer meaningful clinical value and emphasize the need for larger, multi-center studies.

Statistical analysis

Statistical analysis was performed using IBM SPSS Statistics, version 26.0 (IBM Corp., Armonk, NY). Descriptive statistics were utilized to summarize patient characteristics. Chi-square tests were employed to assess associations between categorical variables, with a significance level set at p < 0.05. For all significant associations, odds ratios (ORs) with 95% confidence intervals (CI) were calculated to quantify effect sizes. To account for multiple comparisons and reduce the risk of type I error, Bonferroni correction was applied by adjusting the significance threshold to p < 0.005 (0.05/10) for the primary outcome analyses involving multiple predictors of visual recovery. Factors affecting vision outcomes were analyzed, including initial visual acuity, duration of ON, and treatment type.

## Results

Demographic characteristics

The results in Table 1 present the demographic characteristics of the sample. The results revealed that 60.0% were between 16 and 26 years old, 27.5% were between 27 and 38 years old, and 12.5% were 39 years or older. Regarding gender, 20.0% were male, and 80.0% were female. In terms of nationality, 82.5% were Saudi, and 17.5% were non-Saudi. Regarding smoking habits, 5.0% were smokers, and 95.0% were non-smokers. Finally, concerning chronic health problems, 80.0% of participants reported no such issues, while 20.0% indicated having chronic health problems (hypertension, diabetes mellitus, and autoimmune disorders). These results demonstrate a predominance of younger, female, Saudi, non-smoking individuals without chronic health problems in the sample.

**Table 1:** Demographic characteristics of the sample

Variables	Characteristics	Frequency	Percentage
Age	16-26 years	24	60.0
	27-38 years	11	27.5
	39 years and older	5	12.5
Gender	Male	8	20.0
	Female	32	80.0
Nationality	Saudi	33	82.5
	Non-Saudi	7	17.5
Smoking	Smoker	2	5.0
	Non-smoker	38	95.0
Chronic health problems	No	32	80.0
	Yes	8	20.0

Clinical data

The results in Table 2 showed that most cases of ON were idiopathic (52.5%) or associated with MS (45.0%), with a small percentage linked to NMOSD (2.5%). In terms of laterality eye involvement), most cases were unilateral (92.5%), while only a few were bilateral (7.5%). Regarding the number of ON episodes, most individuals experienced one episode (77.5%), while fewer had two episodes (12.5%) or three or more episodes (10.0%). In terms of duration, the majority of patients had ON for one to 10 days (90.0%), with a small percentage having it for 21 to 25 days (5.0%) or more than 25 days (5.0%).

**Table 2:** Clinical data

Variables	Characteristics	Frequency	Percentage
Cause of optic neuritis	Idiopathic	21	52.5
	Multiple sclerosis (MS)	18	45.0
	Neuromyelitis optica spectrum disorder (NMOSD)	1	2.5
Laterality (eye involvement)	Unilateral	37	92.5
	Bilateral	3	7.5
Number of optic neuritis episodes	1	31	77.5
	2	5	12.5
	3 or more	4	10.0
Duration of optic neuritis (days)	1-10 days	36	90.0
	21-25 days	2	5.0
	More than 25 days	2	5.0

Clinical signs and symptoms

**Figure 1:**
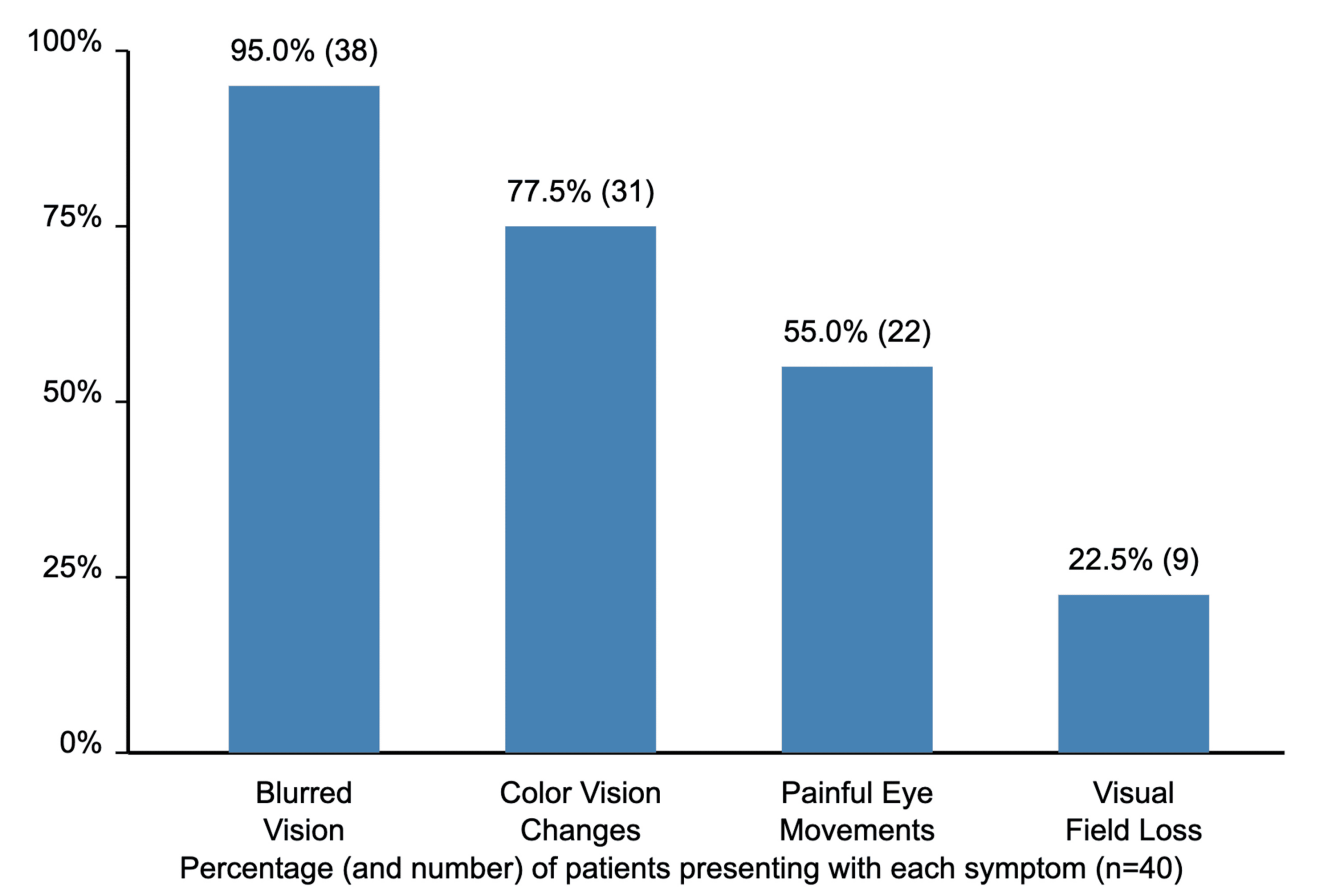
Clinical signs and symptoms

In this study, the symptoms experienced by participants included blurred vision (n = 38, 95%) of participants, color vision changes (n = 31, 77.5%), painful eye movements (n = 22, 55%), and visual field loss (n = 9, 22.5%). These percentages indicate that blurred vision and color vision changes are the most commonly reported symptoms among the group, while visual field loss is the least frequent (see Figure 1).

Associated neurological symptoms or signs 

In a study examining neurological symptoms and signs, the results presented in Table 3 show that out of the participants, 80% exhibited a defective pupillary reaction, indicating a significant impairment, while only 20% had a normal pupillary response. When assessing painful eye movement, 45% had no painful eye movement, whereas 55% experienced painful eye movements. In terms of color vision, 77.5% had decreased color vision, suggesting a notable vision impairment, and only 22.5% had normal color vision. Regarding initial visual acuity, 40% had an acuity greater than 20/200, indicating relatively good vision, while 50% had an acuity of less than 20/200, signifying severe visual impairment, and 10% had exactly 20/200, a threshold for significant vision loss. For the optic disc examination, 37.5% had a blurred optic disc, 45% had a normal optic disc, and 17.5% had a pale optic disc, indicating varying degrees of optic nerve damage. Brain MRI findings revealed demyelinating lesions in 50% of the participants, while the other 50% showed no lesions, highlighting the presence of neurological changes in half of the participants. Lastly, MRI optic enhancement was present in 47.5%, suggesting inflammation or damage to the optic nerves, while 52.5% showed no enhancement, indicating no optic nerve inflammation or damage.

**Table 3:** Associated neurological symptoms or signs

Variables	Characteristics	Frequency	Percentage
Pupillary reaction	Defective	32	80.0
	Normal	8	20.0
Painful eye movement	Absent	18	45.0
	Present	22	55.0
Color vision	Decreased	31	77.5
	Normal	9	22.5
Initial visual acuity	>20/200	16	40.0
	<20/200	20	50.0
	20/200	4	10.0
Optic disc	Blurred	15	37.5
	Normal	18	45.0
	Pale	7	17.5
Brain MRI findings: presence of demyelinating lesions in the brain	Present	20	50.0
	Not present	20	50.0
MRI optic enhancement	Present	19	47.5
	Not present	21	52.5

Regarding the relationship between MRI findings and clinical diagnoses, all patients diagnosed with MS (n = 18, 45%) showed demyelinating lesions on MRI that fulfilled the McDonald criteria. The remaining two patients with demyelinating lesions were classified as idiopathic ON due to insufficient evidence for MS diagnosis. No MS diagnoses were made without supporting MRI evidence. All cases were evaluated by both ophthalmologists and neurologists to ensure diagnostic accuracy. All patients were screened for MOG-IgG antibodies when clinically indicated, but no MOGAD-positive cases were identified. The single NMOSD case was confirmed with positive AQP4-IgG serology. Optic nerve enhancement on MRI was present in 84.2% of MS-associated ON cases and in 38.1% of idiopathic cases.

Treatment and clinical outcome

The treatment outcomes in Table 4 showed that the majority of patients (77.5%) received IV steroids, while 10% received oral steroids, 2.5% were administered immunomodulatory drugs, and 10% did not receive any treatment. Regarding vision outcomes, 80% of patients had vision greater than 20/200, while 20% had vision less than 20/200. Regarding vision prognosis, 37.5% of patients achieved complete resolution of symptoms without residual vision loss, 12.5% had complete resolution with some residual vision loss, 15% continued to experience visual changes, 12.5% had reduced color vision, and 22.5% had missing data.

**Table 4:** Treatment and clinical outcome

Variables	Characteristics	Frequency	Percentage
Treatment received	Immunomodulatory drugs	1	2.5
	IV steroids	31	77.5
	Oral steroids	4	10.0
	None	4	10.0
Vision outcome	>20/200	32	80.0
	<20/200	8	20.0
Vision prognosis	Complete resolution of their symptoms without residual vision loss	15	37.5
	Complete resolution of their symptoms with some residual vision loss	5	12.5
	Continued visual change	6	15.0
	Reduced color vision	5	12.5
	Missing	9	22.5

In this study, 80% of participants (n = 32) experienced an improvement in vision, achieving better than 20/200, indicating significant visual enhancement post-treatment. Conversely, 20% of participants (n = 8) reported a loss of field of vision, highlighting a notable adverse outcome. These findings underscore the treatment's effectiveness in improving visual acuity for the majority, although a minority faced substantial vision-related complications.

Among the 31 patients receiving IV steroids, 25 (80.6%) showed good response with improved visual acuity (>20/200). Six non-responders (19.4%) required extended steroid therapy or oral taper. Plasma exchange was considered for two severe cases but was unavailable at our center, necessitating referral to tertiary centers. During follow-up (mean 8.2 months), seven patients (22.6%) relapsed: five with MS-ON and two with idiopathic ON. Relapsing patients received additional IV steroids, with MS cases referred for immunomodulatory therapy adjustment. Patients with persistent deficits (n = 8) were recommended vision rehabilitation measures.

The results presented in Table 5 indicate no significant differences in laterality based on gender or age group. Among males, 87.5% had unilateral laterality, while 12.5% had bilateral laterality (χ² = 0.630, p = 0.498). For females, 93.8% exhibited unilateral laterality, and 6.3% showed bilateral laterality. In terms of age, 87.5% of individuals aged 16-26 years had unilateral laterality, and 12.5% had bilateral laterality (χ² = 2.162, p = 0.339). All individuals aged 27-38 years and 39 years and older exhibited unilateral laterality, with no cases of bilateral laterality. These results suggest no statistically significant associations between gender, age, and laterality.

**Table 5:** The associations between gender, age, and laterality

Variables	Characteristics	Laterality		χ^2^	p-value
		Unilateral	Bilateral		
Gender	Male	7 (87.5%)	1 (12.5%)	0.630	0.498
	Female	30 (93.8%)	2 (6.3%)		
Age	16-26 years	21 (87.5%)	3 (12.5%)	2.162	0.339
	27-38 years	11 (100%)	0 (0%)		
	39 years and older	5 (100%)	0 (0%)		

Table 6 shows the factors affecting vision outcomes in individuals with ON. The analysis revealed the following: there was no significant association between gender and vision improvement, with 100% of males showing improved vision and 75% of females (χ² = 2.50, p = 0.173). Regarding age, the results show that among participants aged 16-26, 79.2% showed improvement, while 20.8% experienced vision loss. In the 27-38 group, 81.8% improved, and 18.2% had vision loss. For those 39 and older, 80% improved, and 20% experienced vision loss. A chi-square test showed no significant association between age and vision outcomes (χ² = 0.033, p = 0.999). Smoking did not significantly affect outcomes, with 100% of smokers and 78.9% of non-smokers showing improvement (χ² = 0.526, p = 0.468). Those with no chronic health problems had a higher improvement rate (81.3%) compared to those with chronic conditions (75%), though the difference was not significant (χ² = 0.156, p = 0.693). A significant association was found between initial visual acuity and vision outcome, with a chi-square value of 7.5 (χ² = 7.5, p = 0.011, OR = 5.71, 95%, CI: 1.47-22.18). The results showed that patients with an initial visual acuity of >20/200 had a 100% improvement in visual acuity, with no loss of field of vision, indicating that patients with better initial visual acuity were 5.71 times more likely to show improvement compared to those with initial visual acuity <20/200 had a 70% (n = 14) improvement and a 30% (n = 6) loss of field of vision. Patients with an initial visual acuity of 20/200 exhibited a 50% improvement and 50% loss of field of vision. Duration of ON significantly affected outcomes, with 83.3% of those with one to 10 days of symptoms improving, compared to none of those with more than 25 days of symptoms (χ² = 6.030, p = 0.041, OR = 8.33, 95% CI: 1.42-49.01). This indicates that patients presenting within one to 10 days had 8.33 times higher odds of improvement compared to those with prolonged symptom duration. The presence of painful eye movement had no significant effect (χ² = 0.227, p = 0.634), and neither optic disc appearance nor the cause of ON showed significant associations with vision improvement (χ² = 2.623, p = 0.297; χ² = 0.809, p = 0.762). Brain MRI results did not significantly influence outcomes, with 85% of those with demyelinating lesions improving (χ² = 0.625, p = 0.429). The presence of MRI optic enhancement and treatment type also showed no significant effects on vision outcomes (χ² = 0.401, p = 0.527; χ² = 2.021, p = 0.573).

**Table 6:** Factors affecting vision outcomes in individuals with optic neuritis Odds ratios (OR) and 95% confidence intervals (CI) were calculated only for statistically significant findings (p < 0.05). For all non-significant variables (p > 0.05), "not significant" is indicated in the OR column.

Variables	Characteristics	Vision outcome		χ^2^	p-value	OR (95% CI)
		Improved (>20/200)	Loss of field of vision (<20/200)			
Gender	Male	8 (100%)	0 (0%)	2.50	0.173	Not significant
	Female	24 (75%)	8 (25%)			
Age	16-26 years	19 (79.2%)	5 (20.8%)	0.033	0.999	Not significant
	27-38 years	9 (81.8%)	2 (18.2%)			
	39 years and older	4 (80%)	1 (20%)			
Smoking	Smoker	2 (100%)	0 (0%)	0.526	0.468	Not significant
	Non-smoker	30 (78.9%)	8 (21.1%)			
Chronic health problems	No	26 (81.3%)	6 (18.8%)	0.156	0.693	Not significant
	Yes	6 (75%)	2 (25%)			
Initial visual acuity	>20/200	16 (100%)	0 (0%)	7.500	0.011	5.71 (1.47-22.18)
	<20/200	14 (70%)	6 (30%)			
	20/200	2 (50%)	2 (50%)			
Duration of optic neuritis (days)	1-10 days	30 (83.3%)	6 (16.7%)	6.030	0.041	8.33 (1.42-49.01)
	21-25 days	2 (100%)	0 (0%)			
	More than 25 days	0 (0%)	2 (100%)			
Painful eye movement	Absent	15 (83.3%)	3 (16.7%)	0.227	0.634	Not significant
	Present	17 (77.3%)	5 (22.7%)			
Optic disc	Blurred	13 (86.7%)	2 (13.3%)	2.623	0.297	Not significant
	Normal	15 (83.3%)	3 (16.7%)			
	Pale	4 (57.1%)	3 (42.9%)			
Cause of optic neuritis	Idiopathic	16 (76.2%)	5 (23.8%)	0.809	0.762	Not significant
	Multiple sclerosis (MS)	15 (83.3%)	3 (16.7%)			
	Neuromyelitis optica spectrum disorder (NMOSD)	1 (100%)	0 (0%)			
Brain MRI (presence of demyelinating lesions in the brain)	Present	17 (85%)	3 (15%)	0.625	0.429	Not significant
	Not present	15 (75%)	5 (25%)			
MRI optic enhancement	Present	16 (84.2%)	3 (15.8%)	0.401	0527	Not significant
	Not present	16 (76.2%)	5 (23.8%)			
Treatment received	Immunomodulatory drugs	1 (100%)	0 (0%)	2.021	0.573	Not significant
	IV steroids	23 (74.2%)	8 (25.8%)			
	Oral steroids	4 (100%)	0 (0%)			
	None	4 (100%)	0 (0%)			

After applying Bonferroni correction for multiple comparisons, the association between initial visual acuity and vision outcome remained significant (p = 0.011, OR = 5.71, 95% CI: 1.47-22.18), while the association with duration of ON (p = 0.041) should be interpreted with caution as it did not meet the more stringent threshold (p < 0.005). The multiple comparisons adjustment helps control for potential false positive findings when testing numerous variables simultaneously.

## Discussion

This study provides valuable insights into the presentation and outcomes of ON in the Hail region of Saudi Arabia, addressing an important gap in the literature regarding ON manifestation in Middle Eastern populations. The demographic profile of our cohort reveals a strong female predominance (80%) and a primarily young adult population, consistent with global epidemiological patterns documented by Wilhelm and Schabet, who found similar gender disparities in their comprehensive review of ON [8]. The high proportion of Saudi nationals (82.5%) in our sample provides a unique perspective on the condition’s manifestation in this specific population. 

The etiology distribution in our study, with idiopathic cases (52.5%) slightly exceeding MS-associated cases (45%), differs somewhat from Western studies where MS-associated ON is typically more prevalent, as reported by Toosy et al. (2014) [9]. The symptom distribution visualizes the spectrum of ON manifestations; blurred vision (95%) and color vision changes (77.5%) represent the most prominent symptoms, highlighting the significant impact on visual function. These findings reflect the selective neural pathway disruption characteristic of ON [9,10]. Painful eye movements (55%) and visual field loss (22.5%) further demonstrate the condition's neurological complexity. The comprehensive classification of ON diagnostic criteria supports the understanding of these symptoms as reflections of underlying inflammatory processes [10]. The symptom distribution provides valuable regional insights, complementing international research on ON clinical manifestations.

The clinical presentation in our cohort largely mirrors established patterns, with unilateral involvement (92.5%) being the norm. The high prevalence of defective pupillary reactions (80%) and decreased color vision (77.5%) underscores the importance of these clinical signs in diagnosis, consistent with findings from the Optic Neuritis Treatment Trial (ONTT) follow-up studies [11]. Similar studies have reported significant neurological manifestations in Saudi Arabian cohorts, supporting the consistency of these clinical presentations [5].

Neuroimaging findings revealed demyelinating lesions in 50% of participants, which is consistent with the complex relationship between ON and underlying neurological conditions, particularly MS [7]. The presence of MRI optic enhancement in 47.5% of cases provides valuable diagnostic insights into the inflammatory nature of the condition. Our findings on treatment approaches align with established protocols, with IV steroids being the primary intervention (77.5%), consistent with international guidelines [12]. 

The overall positive vision outcomes, with 80% of patients achieving visual acuity better than 20/200, are particularly encouraging and highlight the importance of early intervention. The significant associations between initial visual acuity and duration of symptoms with vision outcomes are crucial clinical insights. After controlling for multiple comparisons, initial visual acuity emerged as the most robust predictor of outcomes. Patients presenting within one to 10 days of symptom onset showed markedly better improvement (83.3%, OR = 8.33, 95% CI: 1.42-49.01), though this finding should be interpreted cautiously due to multiple comparisons, emphasizing the critical nature of timely medical intervention [12]. Similarly, patients with better initial visual acuity (>20/200) demonstrated 5.71 times higher odds of favorable outcomes (95% CI: 1.47-22.18). These findings underscore the importance of rapid diagnosis and treatment in managing ON.

Despite our statistical analysis identifying significant associations only for initial visual acuity and symptom duration, several non-significant findings deserve clinical consideration. Higher improvement rates observed in males (100%) compared to females (75%) suggest potential sex-based differences in recovery patterns. Patients with normal optic disc appearance showed better outcomes (83.3%) than those with pale discs (57.1%), and MS-associated ON cases demonstrated slightly better improvement rates (83.3%) compared to idiopathic cases (76.2%). These trends, while not statistically significant, may inform clinical decision-making and highlight areas for future research with larger sample sizes.

While our study provides significant regional insights into ON, it also establishes important associations between early presentation and favorable outcomes, contributing to the understanding of ON and highlighting region-specific patterns. However, several limitations should be acknowledged. The relatively small sample size and single-center design may limit the generalizability of our findings. Future multi-center studies with larger cohorts are needed to offer a more comprehensive understanding of ON in the Saudi population.

## Conclusions

This study examines ON in Hail, Saudi Arabia, revealing a significant predominance of young females, with many cases being idiopathic or linked to MS. Early intervention with intravenous steroids proved effective, resulting in improved visual outcomes for 80% of patients. The findings emphasize the association between initial visual acuity, symptom duration, and treatment success, informing best practices for patient care in this region. By providing insights specific to this region, the research enhances understanding of ON and informs clinical practices aimed at improving patient care and outcomes.

## References

[1] Deschamps R, Lecler A, Lamirel C (2017). Etiologies of acute demyelinating optic neuritis: an observational study of 110 patients. Eur J Neurol.

[2] Hickman SJ, Petzold A (2022). Update on optic neuritis: an international view. Neuroophthalmology.

[3] Vanikieti K, Janyaprasert P, Lueangram S (2020). Etiologies of acute optic neuritis in Thailand: an observational study of 171 patients. Clin Ophthalmol.

[4] Greco G, Colombo E, Gastaldi M, Ahmad L, Tavazzi E, Bergamaschi R, Rigoni E (2023). Beyond myelin oligodendrocyte glycoprotein and aquaporin-4 antibodies: alternative causes of optic neuritis. Int J Mol Sci.

[5] Alturki YM, Jawa HT, Alghamdi GA (2024). Clinical outcomes of optic neuritis: a retrospective study at a tertiary medical center in Saudi Arabia. Neurosciences (Riyadh).

[6] Behbehani R, Ali A, Alakool A, Farouk S, Alroughani R (2024). The clinical profile of new-onset optic neuritis in arabs, a tertiary center experience in Kuwait. Heliyon.

[7] Braithwaite T, Subramanian A, Petzold A (2020). Trends in optic neuritis incidence and prevalence in the UK and association with systemic and neurologic disease. JAMA Neurol.

[8] Wilhelm H, Schabet M (2015). The diagnosis and treatment of optic neuritis. Dtsch Arztebl Int.

[9] Toosy AT, Mason DF, Miller DH (2014). Optic neuritis. Lancet Neurol.

[10] Petzold A, Fraser CL, Abegg M (2022). Diagnosis and classification of optic neuritis. Lancet Neurol.

[11] Beck RW, Cleary PA, Backlund JC (1994). The course of visual recovery after optic neuritis. Ophthalmology.

[12] Hajjar A, Jacob A, Smith S, Eldweik L (2024). Features and associations of optic neuritis in the Middle East: a cross-sectional study. AJO Int.

